# Block of Voltage-Gated Sodium Channels by Aripiprazole in a State-Dependent Manner

**DOI:** 10.3390/ijms232112890

**Published:** 2022-10-25

**Authors:** Karl Josef Föhr, Michael Rapp, Michael Fauler, Thomas Zimmer, Bettina Jungwirth, David Alexander Christian Messerer

**Affiliations:** 1 Department of Anesthesiology and Intensive Care Medicine, University Hospital Ulm, 89081 Ulm, Germany; 2 Institute of General Physiology, University of Ulm, 89081 Ulm, Germany; 3 Institute of Physiology, University Hospital of Jena, 07747 Jena, Germany; 4 Department of Transfusion Medicine and Hemostaseology, Friedrich-Alexander University Erlangen-Nuremberg, University Hospital Erlangen, 91052 Erlangen, Germany

**Keywords:** aripiprazole, sodium channel, cardiotoxicity, electrophysiology, patch clamp

## Abstract

Aripiprazole is an atypical antipsychotic drug, which is prescribed for many psychiatric diseases such as schizophrenia and mania in bipolar disorder. It primarily acts as an agonist of dopaminergic and other G-protein coupled receptors. So far, an interaction with ligand- or voltage-gated ion channels has been classified as weak. Meanwhile, we identified aripiprazole in a preliminary test as a potent blocker of voltage-gated sodium channels. Here, we present a detailed analysis about the interaction of aripiprazole with the dominant voltage-gated sodium channel of heart muscle (hNa_v_1.5). Electrophysiological experiments were performed by means of the patch clamp technique at human heart muscle sodium channels (hNa_v_1.5), heterologously expressed in human TsA cells. Aripiprazole inhibits the hNa_v_1.5 channel in a state- but not use-dependent manner. The affinity for the resting state is weak with an extrapolated K_r_ of about 55 µM. By contrast, the interaction with the inactivated state is strong. The affinities for the fast and slow inactivated state are in the low micromolar range (0.5–1 µM). Kinetic studies indicate that block development for the inactivated state must be described with a fast (ms) and a slow (s) time constant. Even though the time constants differ by a factor of about 50, the resulting affinity constants were nearly identical (in the range of 0.5 µM). Besides this, aripirazole also interacts with the open state of the channel. Using an inactivation deficit mutant, an affinity of about 1 µM was estimated. In summary, aripiprazole inhibits voltage-gated sodium channels at low micromolar concentrations. This property might add to its possible anticancer and neuroprotective properties.

## 1. Introduction

Voltage-gated sodium channels (VGSCs) are composed from one out of nine different pore forming α-subunits which are associated with no, one or two out of four auxiliary ß-subunits [1,2]. The α-subunits are designated as Na_v_1.1 to Na_v_1.9 according to their phylogeny. Other diversity arises from alternative splicing of mRNA and post-translational modifications. The distribution of the different subunits is tissue-specific. Na_v_1.4 and Na_v_1.5 are predominantly found in skeletal and heart muscle cells, whereas Na_v_1.1 to Na_v_1.3 or Na_v_1.8 occur in neurons. According to pharmacological parameters the individual channels are classified as TTX-sensitive or TTX-resistant, depending on the concentration of tetrodotoxin (TTX), which is required for their blockage. Further classifications are based on different subunit specific electrophysiological properties [3].

Aripiprazole is an atypical antipsychotic drug, which has initially been approved for the treatment of schizophrenia and severe manic episodes in bipolar disorder [4]. Meanwhile, the list includes further indications. Studies show its efficacy to treat tic disorders, post-traumatic stress disorder, autism, and depression [5,6,7,8]. Aripiprazole belongs to the second generation of antipsychotics with a unique pharmacological profile, termed polypharmacology, indicating that aripiprazole might interact in different ways with various receptors. Most prominent here is its interaction with dopamine D_2_ receptors. These receptors play a crucial role for antipsychotics. Thus far, all existent antipsychotics have produced extrapyramidal side effects as they were dopamine D_2_ antagonists. Consequently, aripiprazole has been designed as the first antipsychotic drug with a D_2_ agonistic property [9]. To this end, aripiprazole was engineered from quinolinone, a known agonist of D_2_-receptors (previously termed dopamine autoreceptors), which served as a lead compound [4,10,11]. Other receptors that are affected by aripiprazole are dopamine D_3_ and serotonin 5-HT_1A_ receptors, which are activated, while 5-HT_2A_ receptors are antagonized [12,13]. Taken together, all these properties also extended the application area of aripiprazole. In this way, aripiprazole was suggested to reduce antipsychotic-induced hyperprolactinemia [14,15]. Furthermore, great importance is attached to its possible anticancer and neuroprotective properties [16,17,18,19]. Regarding side effects, aripiprazole is rated to produce less kinetic disorders compared to other antipsychotics [9]. In therapeutic concentrations, it also seems to cause less cardiovascular adverse reactions than other antipsychotics [20,21,22]. Concerning sedative or weight-gaining effects, controversial results have been reported [5,23].

The main targets for aripiprazole are G-protein coupled receptors, while its effects on ligand-gated ion channels appear to be weak [13]. Interactions with voltage-gated channels have, to the best of our knowledge, not been studied with the exception of a voltage-gated potassium channel [24]. Chemically related drugs such as nefazodone [25] and trazodone [26] as well as structurally similar atypical anti-psychotics such as risperidone [27] and iloperidone [28] display well-documented effects on various voltage-gated channels. In the present work, we tested the hypothesis that aripiprazole might functionally interact with hNa_v_1.5, which is of special interest given recent reports of cardiotoxicity upon aripiprazole overdose [21,29] and the involvement of hNa_v_1.5 in the malignancy of distinct cancers [19,30,31].

## 2. Results

VGSCs may exist in different states (resting, open, inactivated), which are achieved by conformational changes [1]. Under physiological conditions, none of the different states exists exclusively. Moreover, the relative proportion of the different states varies in a voltage- and time-dependent manner. The patch clamp technique is a widely used electrophysiological method, which allows to a great extent to force the channels into the individual states, whereby the interaction of the drug with each state and the impact on the corresponding transitions can be analyzed in detail [1,32,33].

### 2.1. Aripiprazole Blocks hNa_v_1.5 Channels

**Figure 1:**
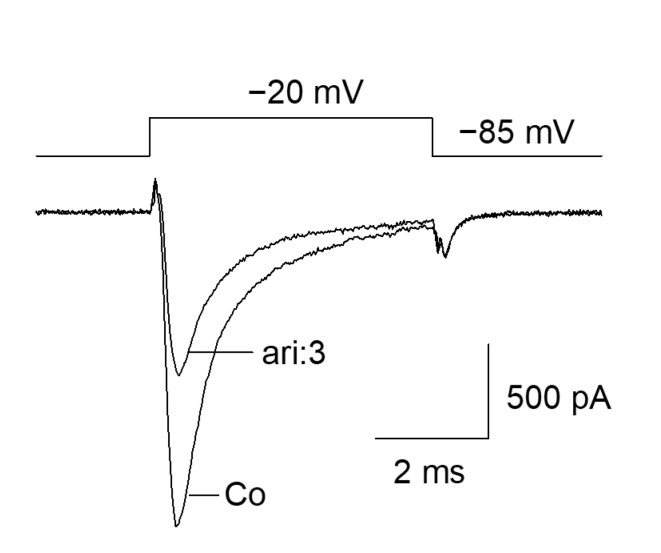
Inhibition of hNa_v_1.5 channels by aripiprazole: Representative current traces obtained for control (Co) and in the presence of 3 µM aripiprazole (ari: 3). Channel activations were carried out by short depolarizations to −20 mV for 5 ms from a holding potential of −85 mV.

The fundamental capability of aripiprazole to block VGSCs was shown as the outcome of a preliminary experiment (Figure 1). Starting from a physiological holding potential of −85 mV, hNa_v_1.5 transfected TsA201 cells were depolarized to −20 mV for 5 ms. In the absence of aripiprazole, a typical fast inactivating inward current was observed, which was reduced to about half of its size in the presence of 3 µM aripiprazole (Figure 1). This figure is an illustration to visualize the effect of aripiprazole at an about half-maximal inactivation. An accumulation of fast inactivation is excluded as all experiments were started with repeated activations in the absence of the drug where no decline in current amplitude was observed.

### 2.2. Interaction with the Resting State

**Figure 2:**
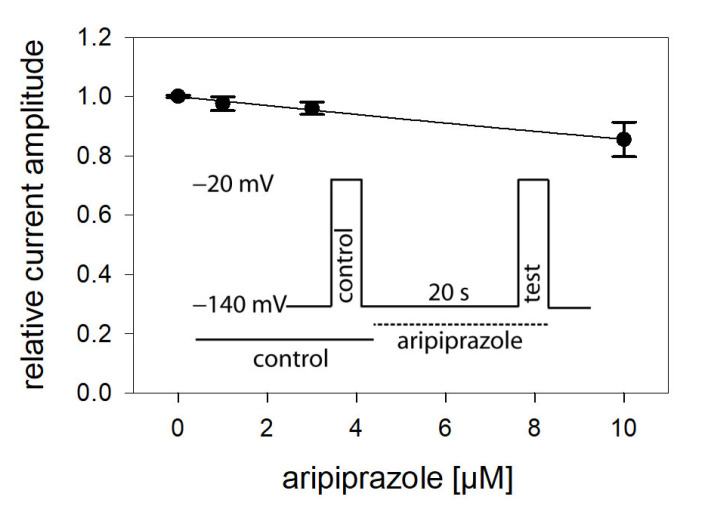
Interaction with the resting state. Relative current amplitudes versus the concentration of aripiprazole are illustrated. Channel activations in the absence (control) and presence of different concentrations of aripiprazole (test) were carried out from a holding potential of −140 mV (insert). Current amplitudes obtained for the test pulses were related to their controls and plotted against the concentration of aripiprazole. Data points were fit to Equation (1). Note, even at the highest concentration of aripiprazole (10 µM), the inhibitory effect small. Thus, the calculated affinity (K_r_: 54.7 ± 13.0 µM) has to be considered as an approximate estimation.

In view of a possible state-dependent interaction, different parameters were analyzed in more detail, starting with the interaction with the resting state. To this end, a double pulse protocol, as illustrated by the insert of Figure 2, was carried out in the absence and presence of different concentrations (1–10 µM) of aripiprazole. Accordingly, channel activations were carried out by brief (5 ms) depolarizations to −20 mV outgoing from the holding potential of –140 mV. For evaluation, current amplitudes in the presence of aripiprazole were related to their respective controls and plotted against the concentration of aripiprazole (Figure 2). As it is evident, the inhibitory effect of aripiprazole was small, even at the highest concentration tested (10 µM). Therefore, the calculated affinity for the resting state (K_r_) can only be considered as a rough estimation. Using Equation (1), half-maximal inhibition is calculated to occur at 54.7 ± 13.0 µM aripiprazole. As the inhibitory potency of aripiprazole was strongly diminished when the channels were activated from a very negative holding potential (−140 mV), it is obvious that aripiprazole interacts with the hNa_v_1.5 channel in a strong voltage-dependent manner.

### 2.3. Voltage Dependence of Activation

**Figure 3:**
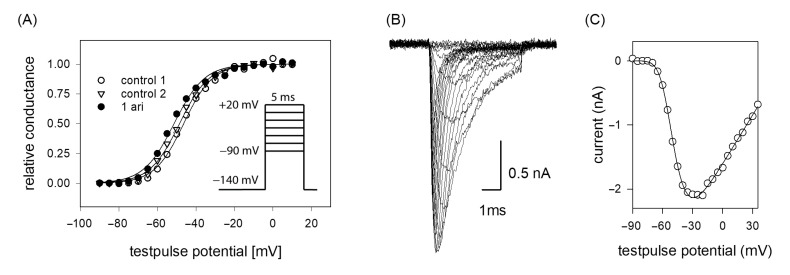
(**A**) Voltage dependence of activation. The graph illustrates representative data obtained from one typical cell. Normalized peak conductances in the absence (control 1 and 2; open circles and triangles) and presence of 1 µM aripiprazole (filled circles) versus test pulse potential are shown. The potential difference in activation midpoints between control 1 and 2 was taken into account as the drug-independent shift. Altogether, mean half-maximal activation for control 1 and 2 occurred at –43.6 ± 3.0 mV and −45.2 ± 3.4 mV with a mean slope of 6.5 mV. The corresponding values in the presence of 1 µM aripiprazole were −46.5 ± 4.2 mV, slope 6.7 mV. Insert: Experimental scheme. Starting from a holding potential of −140 mV, test pulses for 5 ms to potentials ranging from −90 to +20 mV (increment 5 mV) were carried out. Interpulse interval was 5 s. (**B**) Overlay of original current traces (control 1) obtained for the data shown in (**C**) Peak current amplitudes at different test pulse potentials from the traces shown in (**B**). All data are from the same cell.

If the efficacy of a drug interaction happens in a voltage-dependent manner, this may affect all processes/transitions, which reveal a potential dependency, most prominently channel activation and inactivation. In a first set of experiments, voltage-dependency of channel activation was analyzed. To this end, brief depolarizations from a holding potential of −140 mV to different test pulse potentials (range: −90 to +20 mV in steps of 5 mV) were carried out. Plotting the resulting current amplitudes versus the test pulse potential resulted in a biphasic curve with a maximum value due to the overlap of two contrary processes. In the first part, current amplitudes increased after having reached a threshold value with more positive test potentials as more channels become activated. In the second part, the current amplitudes declined as the test pulse potentials approached more and more the equilibrium potential for sodium ions (Figure 3B,C). For ease of evaluation, the changes in driving force were considered according to Equation (2) whereby the potential dependent behavior can be described with a single Boltzmann function (Figure 3A). From the fit of data via Equation (3), the potential of half-maximal activation (activation midpoint) and the corresponding slope resulted. If the inhibitory effect of aripiprazole was based on an interaction with the process of channel activation, a shift of the activation curve to more positive potentials would be expected, indicating that in the presence of aripiprazole stronger depolarizations would be required compared to control. However, this does not apply here. In order to consider the well-known drug independent shift, initially two activation curves were registered in the absence of drug (control 1, control 2). From the potential difference of their activation midpoints, the drug-independent shift was calculated for each cell and subtracted from the shift obtained between control 2 and aripiprazole. All consecutive activation curves (between controls or between control and drug) were carried out in identical time intervals. Overall, the midpoints of the activation curves averaged for the first and second control at −43.6 ± 3.0 mV and −45.2 ± 3.4 with a slope of 6.5 ± 0.8 mV. The corresponding values in the presence of aripiprazole (1 µM) were −46.5 ± 4.1 mV with a slope of 6.7 ± 0.6 mV. The linear drift of V_50_ from the first over the second control to aripiprazole averaged to −1.40 ± 0.54 mV (*p* = 0.01), while 1 µM aripiprazole had no significant independent additional effect (0.03 ± 0.94 mV, *p* = 0.97). Thus, the inhibitory impact of aripiprazole on the hNa_v_1.5 does not seem to arise from an interaction with the process of channel activation.

### 2.4. Voltage Dependence of Fast Inactivation

**Figure 4:**
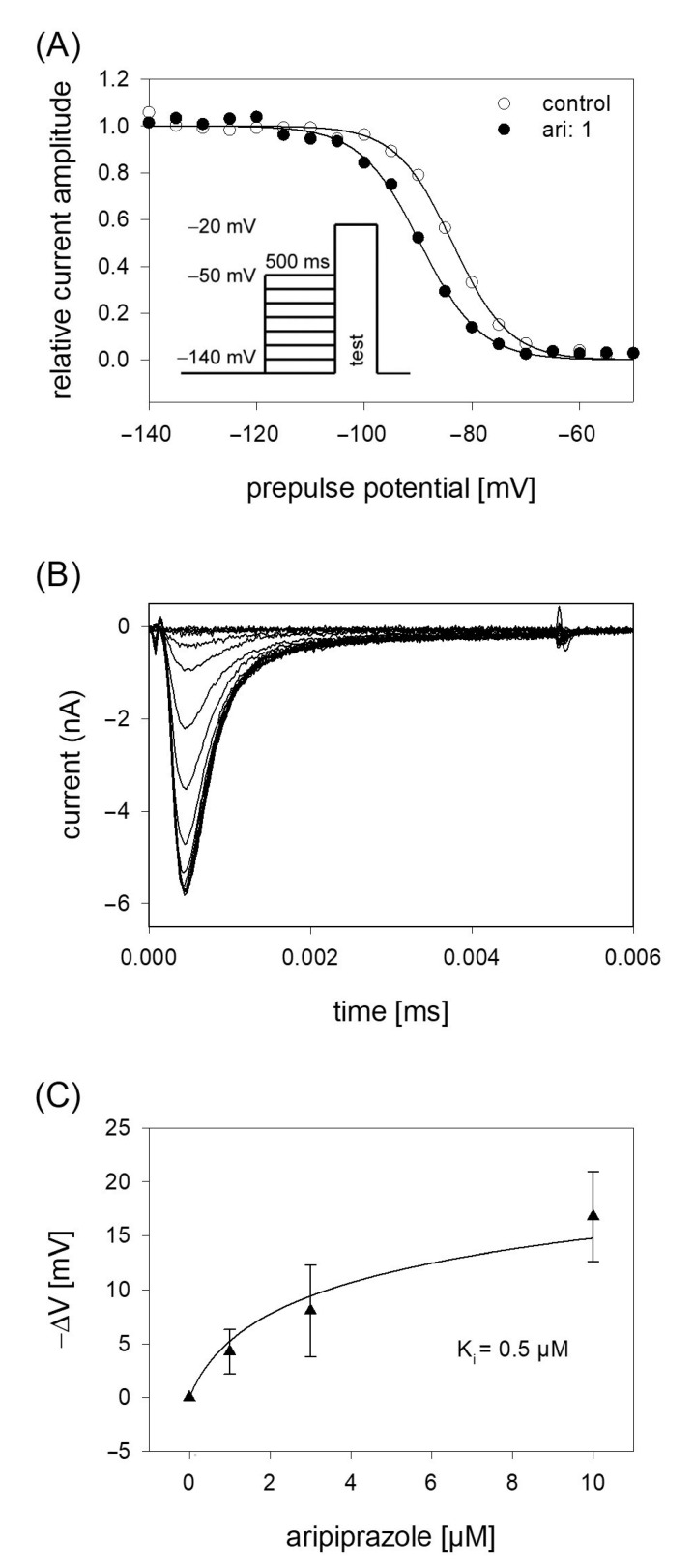
Voltage dependence of fast inactivation. (**A**) Data of normalized peak currents are plotted against the prepulse potential. Solid lines represent fits according to Equation (4). Insert: Experimental scheme. Voltage dependence of fast-inactivation was determined by measuring sodium currents elicited by 5 ms depolarizations to −20 mV after conditioning prepulses for 500 ms to different potentials (range −140 to −50 mV) in the absence (open circles) and presence (filled circles) of 1 µM aripiprazole. Holding potential was −140 mV. (**B**) Overlay of original current traces obtained for control. (**C**) Relative shift of inactivation midpoints in relation to the applied concentration of aripiprazole. Data are corrected for the drug-independent shift (1.0 mV/measuring cycle) and fitted according to Equation (6) using the mean slope obtained in the presence of aripiprazole. The fit gives an affinity of 0.5 ± 0.14 µM for the fast-inactivated state.

In order to analyze potential dependency of fast inactivation, the following experimental scheme was used. Starting from a holding potential of −140 mV, prepulses for 500 ms to different potentials (range −140 to −50 mV; increment 5 mV) were applied immediately before the test pulse to −20 mV was carried out (insert Figure 4A). In this way, the amount of inactivated channels increased with more positive prepulse potentials. For evaluation, all current amplitudes were normalized to the largest current amplitude (obtained from the most negative prepulse potentials) and plotted versus the prepulse potential. Data were fitted according to Equation (4) in order to generate inactivation curves. The most important parameters here were the potential at which half of the channels were inactivated, and the slope of the curve. If aripiprazole interacts with the process of fast inactivation, a shift of the inactivation curve to more negative potentials is expected. As illustrated by Figure 4A, aripiprazole leads to a prominent shift of the inactivation curve to more negative potentials. In particular, mid-points of the inactivation curves were shifted from −85.2 ± 2.4 mV for control to −90.1 ± 2.1 mV in the presence of 1 µM aripiprazole. With higher concentrations of aripiprazole, the shift became even stronger. In particular, for 3 and 10 µM aripiprazole, a shift of −9.1 ± 0.5 and −17.8 ± 1.4 mV was estimated, respectively. The slopes for control and in the presence of increasing concentrations of aripiprazole were 4.6 ± 0.6, 4.6 ± 0.7, 5.1 ± 1.2 and 5.0 ± 0.5 mV, respectively. All data were corrected for the common, drug-independent left shift (mean: −1.01 ± 0.07 mV per measuring cycle, not shown) and plotted versus the concentration of aripiprazole (Figure 4C). From the fit of data according to Equation (6) using the mean slope obtained in the presence of aripiprazole, the affinity for the fast-inactivated state K_i_ was calculated to be 0.50 ± 0.14 µM.

### 2.5. Interaction with the Slow Inactivated State

**Figure 5:**
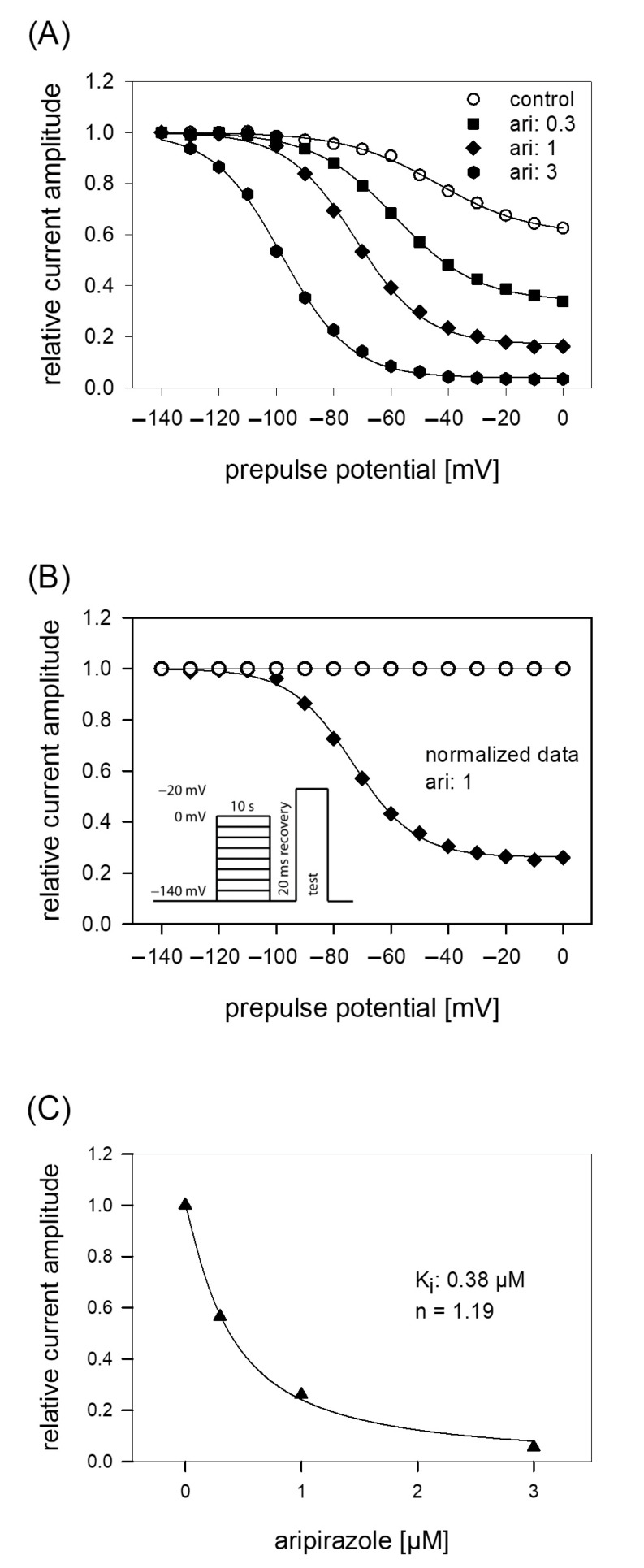
Voltage dependence of slow inactivation. (**A**) Data of normalized peak currents from a representative cell are plotted versus the prepulse potential. Solid lines are fits according to Equation (5) for control (open circles) and different concentrations of aripiprazole (filled symbols). The experimental scheme as illustrated by the insert of B consisted of channel activations to −20 mV after long-lasting (10 s) conditioning prepulses to different potentials and a short (20 ms) recovery period at −140 mV immediately before the test pulse. (**B**) Normalized data for 1 µM aripiprazole were obtained by dividing the values obtained in the presence of 1 µM aripiprazole by those obtained for control. It is evident that current amplitudes continuously decreased with an increase in prepulse potential. (**C**) Concentration response curve taken from normalized current amplitudes obtained at the prepulse potential of 0 mV. The affinity to the slow inactivated state (K_i_) obtained from this experiment is given in the insert and was obtained from a fitting of data according to Equation (1). Altogether, an affinity for the slow inactivated state of 0.39 ± 0.07 µM resulted.

The protocol for analyzing the interaction with the slow inactivated state differs in two points from that of fast-inactivation. First, the duration of the prepulse was prolonged to 10 s. Second, immediately before the test pulse a short recovery period (20 ms, −140 mV) was inserted to eliminate or minimize the contribution from fast-inactivation (see insert to Figure 5B). For evaluation, all current amplitudes were normalized with respect to the largest amplitude and plotted versus the prepulse potential (Figure 5A). Data were fitted with Equation (5), respecting an incomplete inactivation. Under control, half-maximal inactivation occurred at −47.0 ± 8.7 mV with a slope of 19.5 ± 3.9 mV. At a prepulse potential of 0 mV, the remaining current amplitude amounted to 55.6 ± 11%. In order to respect the current decline under control in the potential dependent behavior of aripiprazole, data were normalized with respect to control by dividing the values obtained in the presence of aripiprazole by those obtained for control (Figure 5B). Normalized data for 1 µM aripiprazole are shown. It is evident that current amplitudes continuously decreased with an increase in prepulse potential. In the presence of aripiprazole, the inactivation curve shifted to more negative values and the remaining current at 0 mV decreased (Figure 5A). For 0.3, 1, and 3 µM aripiprazole half-maximal inactivation occurred at −56.7 ± 6.5 mV, −72.4 ± 6.3 mV and −98.2 ± 3.8 mV, respectively. Corresponding slopes were 17.0 ± 3.0 mV, 15.3 ± 2.5 mV, and 12.8 ± 0.8 mV. For estimating an affinity for the slow inactivated state, normalized current amplitudes obtained at a prepulse potential of 0 mV were used (Figure 5C). Using Equation (1), an affinity of 0.39 ± 0.07 µM was calculated.

### 2.6. Block Development: Kinetic Parameters

**Figure 6:**
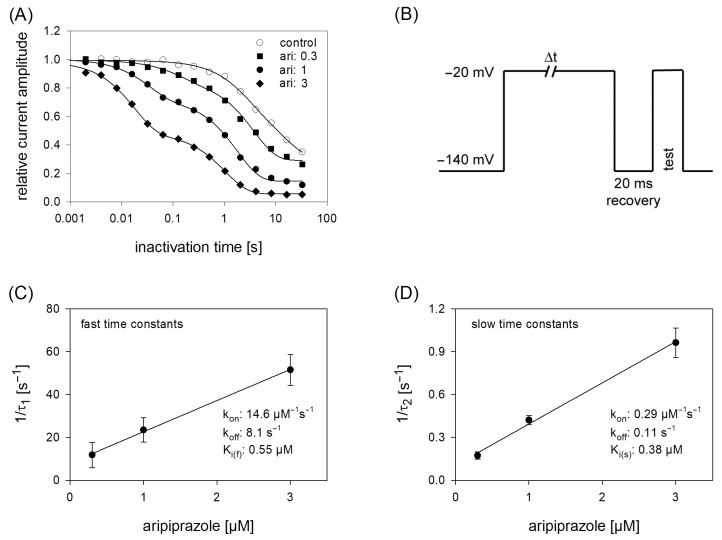
Time course of block development. (**A**) The graph illustrates relative current amplitudes in dependence on the duration of the inactivating prepulse for control (open circles) and in the presence of different concentrations of aripiprazole (filled symbols). Solid lines represent fits of single (control) or double exponential functions (in the presence of aripiprazole). (**B**) Experimental scheme for an individual sweep. Channels were inactivated at −20 mV for a variable duration. Immediately before the test pulse a short recovery period was inserted. The amplitude of the inactivating current was used as control. Interval between individual sweeps was 5 s. (**C**,**D**). The inverse of the time constants of the data shown in A were plotted versus the concentration of aripiprazole. Association (k_on_) and dissociation rate constants (k_off_) were estimated from the slope and y-intercept of the linear fit. Inserts give fit data for this particular experiment. Altogether affinity constants for the fast and slow time constants were 0.55 µM and 0.38 µM, respectively. This evaluation was performed under the reservation that the data from the individual terms were allowed to be handled separately, as mentioned in the discussion.

Due to the prominent interaction of aripiprazole with the inactivated state, further experiments were conducted in this area, first considering kinetic parameters. The most important variable in this kind of experiments is the duration the channels spent in the inactivated state. For experimentation, we used a protocol as outlined by Figure 6B. After an inactivating pulse of variable time (2 ms to 30 s), the test pulse was applied after a short recovery at −140 mV for 20 ms. For evaluation, current amplitudes of the test pulses were related to the size of the inactivating currents and plotted versus the inactivation time. Under control conditions, the current amplitude dropped at inactivation times longer than 100 ms. Here, one exponential function was sufficient to describe the time course. In the presence of aripiprazole, an additional exponential function was required to describe the time course whereby two time constants resulted. With increasing concentrations of aripiprazole, both time constants became faster and the fast time constant gained weight. In case of 1 µM aripiprazole, the fast and slow time constants were 42.0 ± 10.0 ms and 1.95 ± 0.12 s, with the fast component contributing to 33.6 ± 5.5%. To estimate affinity constants from drug-induced changes in kinetic parameters, standard equations have been developed; however, as mentioned in the discussion, these might only provide an approximation with certain limitations. Anyhow, the inverse of the fast and slow time constants were separately plotted versus the concentration of aripiprazole. The on-rates (k_on_) were directly taken from the slope of the linear regression and the off-rates (k_off_) were given by the y-intercepts (Equations (9) and (10)). For the fast time constants, k_on_ and k_off_ amounted to 14.6 µM^−1^s^−1^ and 8.1 s^−1^. The corresponding values for the slow time constants were 0.1µM^−1^s^−1^ and 0.29 s^−1^. From these data, K_i_ values were calculated according to the relation K_i_ = k_off_/k_on_. Even though the fast and slow time constants differed by a factor of about 50, the affinity constants were rather similar, being K_i_fast_ = 0.55 µM and K_i_slow_ = 0.38 µM.

### 2.7. Estimation of Apparent Affinities: Steady-State Parameters

**Figure 7:**
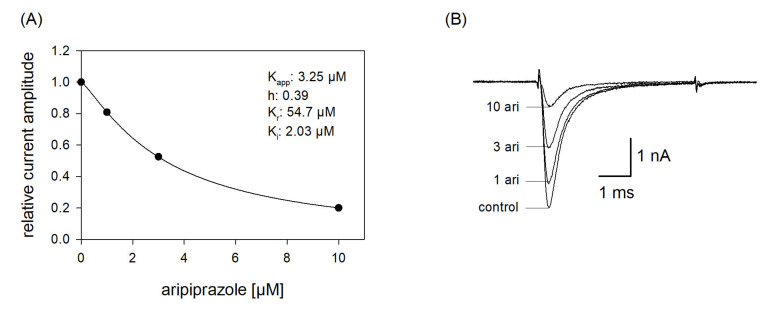
Apparent affinity. (**A**) The graph illustrates relative current amplitudes in the presence of different concentrations of aripiprazole (black circles represent measured normalized current amplitudes, solid line is the result of the fit to Equation (1)). (**B**) Original current traces obtained from channel activations carried out according to the protocol illustrated in Figure 1. The holding potential was set close to half-maximal inactivation (here: −90 mV). Aripiprazole was preincubated for 10 s before the test pulse was given. Interval between individual sweeps was 10 s. The apparent affinity (K_app_) was obtained from a fit of data according to Equation (1). For calculating the affinity to the inactivated state (K_i_), Equation (7) was employed, which respects the affinity to the resting state (K_r_) and the amount (h) of currently available (not-inactivated) channels. Fit parameters for this cell are shown as insert. Altogether, a K_i_ of 2.14 ± 1.16 µM was estimated.

In order to reproduce a more physiological situation, we tested concentration relationships using a holding potential close to half-maximal inactivation. Channel activations were carried out after a preincubation (10 s) with control solution or aripiprazole in different concentrations. From the concentration relationship, half-maximal effective concentrations were calculated using Equation (1) (Figure 7). The resulting affinity constant is termed “apparent affinity” (K_app_), as it relates to the affinity to the different states. Considering that aripiprazole does not interact with the open state (at least with wildtype channels, see below) the inhibition is attributed to an interaction with the resting and the inactivated state. With the knowledge of the affinity for the resting state (K_r_) and the number (h) of available channels (individually estimated for each cell from a preceding inactivation curve), the affinity for the inactivated state was calculated according to Equation (7). Analyzing five cells in this way resulted in a mean K_i_ of 2.14 ± 1.16 µM.

### 2.8. Recovery from Inactivation

**Figure 8:**
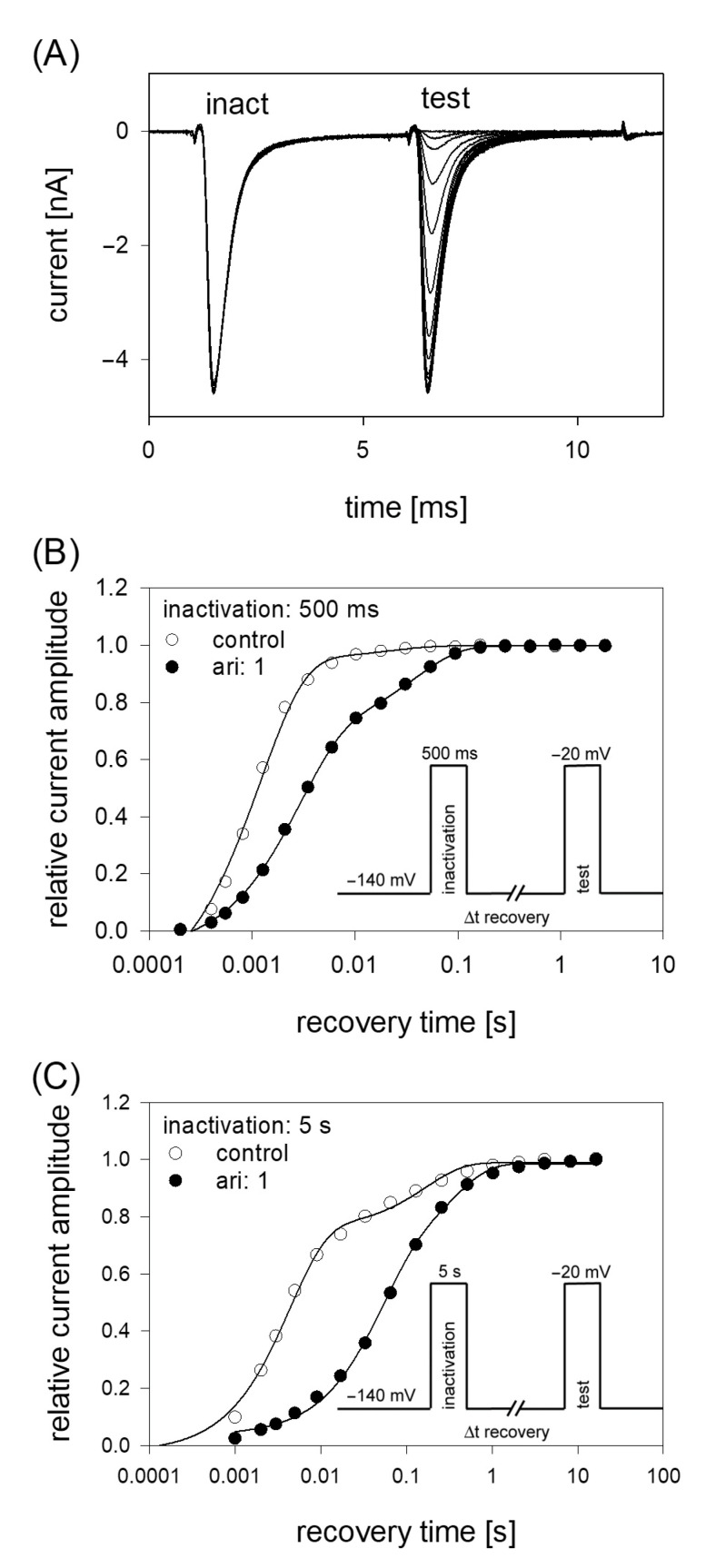
Recovery from inactivation. (**A**) Overlay of 19 original current traces obtained for control from the initial part of the conditioning inactivation pulses (inact.), which served as control and their corresponding test pulses (test). The variable recovery time is not illustrated. (**B**) Recovery from fast inactivation was performed using an inactivation time of 500 ms. The relative amount of available channels expressed as relative current amplitude (test/inact.) versus the recovery time at −140 mV is illustrated. The pulse protocol is illustrated by the inset. Interval between individual sweeps was 10 s and aripiprazole was applied at 1 µM. Solid lines represent fits with two exponential functions according to Equation (11). (**C**) Otherwise identical protocol as in (**B**), but with the inactivation time set to 5 s.

If a drug interacts with the inactivated state of an ion channel, the relative number of channels staying in the inactivated state will be increased. Thus, a new equilibrium between the different states will arise which might include a facilitated transition of channels into the inactivated state. Consequently, the transition out of the inactivated state (recovery) will be retarded in the presence of the drug. The latter process can be investigated by estimating time constants for recovery from inactivation. To this end, a double pulse protocol using two different durations for the inactivating pulse (500 ms and 5 s) was employed. The protocol consisted of an initial inactivating pulse, which also served as control. Thereafter the membrane was held for a variable time at resting potential (−140 mV) before the test pulse was applied (Figure 8). The amplitudes of the test pulses were related to the corresponding control (here: size of the inactivating pulse) and plotted versus the recovery time. Data fitting was performed using two exponential functions (Equation (11)). After an inactivation lasting for 500 ms, most channels (95.2 ± 3.8%) recovered under control with a time constant τ_1_ = 1.6 ± 0.5 ms; the remainder with τ_2_ = 29.6 ± 10.9 ms. In the presence of 1 µM aripiprazole, the fast and slow time constants increased to 2.9 ± 0.8 ms and 35.4 ± 4.5 ms, respectively. More prominently, the fraction of fast recovering channels decreased to 55.0 ± 22.7% while that of slower recovering channels increased inversely (Figure 8). Using an inactivation time of 5 s, the fast time constant increased from 2.5 ± 1.2 ms for control to 47.9 ± 45.3 ms in the presence of 1 µM aripiprazole. The corresponding values for the slow time constant were 92.0 ± 50.4 ms and 431.3 ± 372.2 ms. Here, the relative proportions for the fast and slow component did not change as much as observed before for the short inactivation time. In particular, the relative amount of fast time constant decreased from 73.1 ± 12.3% under control to 64.6 ± 22.5% in the presence of 1 µM aripiprazole.

### 2.9. Interaction with the Open State

**Figure 9:**
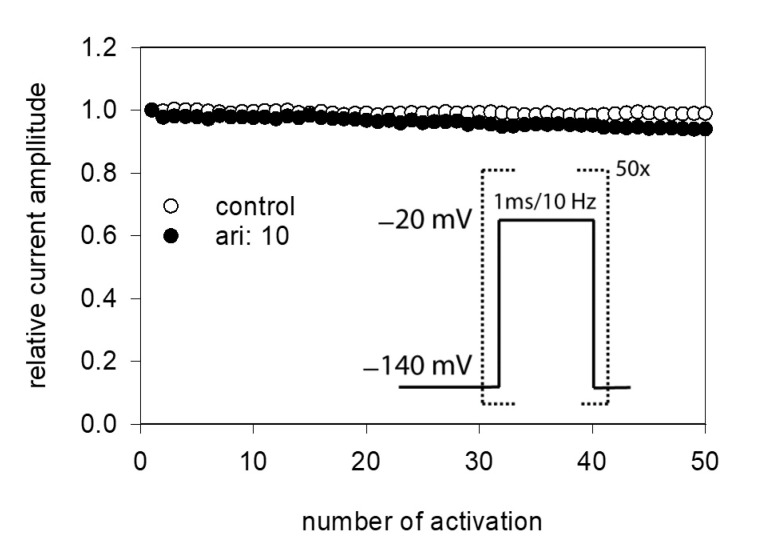
Analysis for use-dependence. Short activations (1 ms) were carried out at a frequency of 10 Hz in the absence and presence of 10 µM aripiprazole. Current amplitudes declined under control from the first to the last activation (50th pulse) by 1.8 ± 1.1%. In the presence of aripiprazole (10 µM), current reduction amounted to 8.2 ± 2.1%. Aripiprazole was preincubated for 20 s.

Due to the very short open time of VGSCs (about 1 ms), a drug interaction with this state is seldom observed upon a single activation. Therefore, repeated activations were carried out at increased frequency to increase the overall open time. In order to minimize channel inactivation time during each activation cycle, the activation time was kept short. For these experiments, the activation time was set to 1 ms. All experiments were started with a repeated low-frequency stimulation (0.2 Hz) until a stable current amplitude was achieved. Control or drug solution in different concentrations was applied for 20 s before the high-frequency stimulation (10 Hz) was executed. For evaluation, all current amplitudes were related to the amplitude of the first response of the high-frequency stimulation train. For control, a minimal decline (1.8 ± 1.1%) in the current amplitude from the first to the last (50th) activation was observed. As there was also virtually no use-dependency in the presence of 1 µM aripiprazole, we increased its concentration to 10 µM (Figure 9). Even with this high concentration, there was only a small current reduction of 8.2 ± 2.1%. From this minimal current reduction, it is concluded that aripiprazole does not operate in a relevant use-dependent manner.

**Figure 10:**
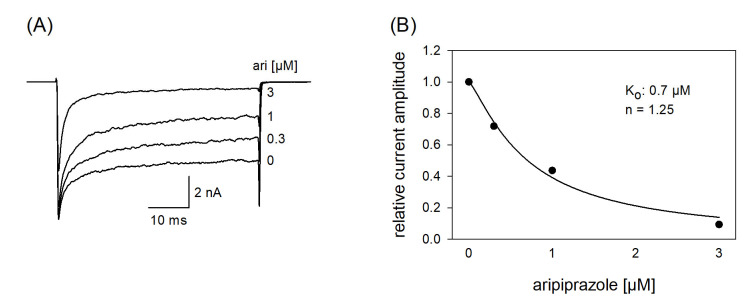
Interaction with the open state. (**A**) Original current traces obtained from the inactivation deficient mutant Nav1.5_CW in the absence and presence of different concentrations of aripiprazole. Individual activations were carried out from a holding potential of −140 mV by stepping to −20 mV for 50 ms. Drugs were preincubated for 60 s before individual activations were carried out. (**B**) For evaluation, relative plateau current amplitudes (measured at the end of the depolarization) were plotted versus the concentration of aripiprazole (the amplitude obtained for control was set to 1). Data fitting according to Equation (1) revealed fit parameters of this experiment as illustrated. K_o_ indicates the affinity for the open state. Altogether, an affinity for the open state of 0.94 ± 0.25 µM was estimated.

In a next set of experiments, we tested a channel mutant with reduced capability to inactivate (hNa_v_1.5_L409C_A410W; CW mutant [34]). With this mutant, the channels stay open to a large extent even after a prolonged activation (50 ms). If a drug interacts with the open state of a channel, a concentration-dependent reduction of the non-inactivating plateau current is expected. Original traces from these experiments are illustrated by Figure 10A. It is evident that the plateau current, measured at the end of the 50 ms lasting depolarization, was diminished in a concentration-dependent manner with a half-maximal inhibition occurring at 0.94 ± 0.25 µM aripiprazole. Thus, aripiprazole also operates as an open channel blocker.

### 2.10. Binding Site

**Figure 11:**
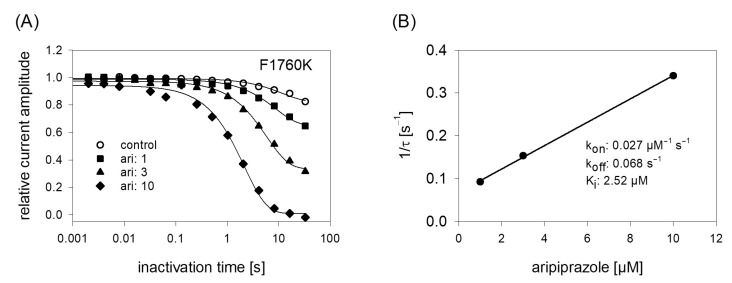
Time course of block development at the F1760K mutant. (**A**) Identical experiments as shown by Figure 6 were carried out with the F1760K mutant. Note: differently to the wildtype, time course of block development for the mutant can be described with one single exponential function. (**B**) Inverse of the time constants versus the concentration of aripiprazole. Listed fit parameters refer to this particular experiment. Altogether, a K_i_ of 2.39 ± 0.28 was estimated.

In order to obtain a hint at which site aripiprazole interacts with the hNa_v_1.5, we made use of an additional mutant (F1760K). The amino acid at this position plays an important role for the interaction with local anesthetics [35]. If a drug interacts at the position 1760, a strongly reduced affinity for the F1760K mutant is expected. In order to compare the effects obtained from the F1760K mutant with data from wildtype, we reused the kinetic protocol (Figure 11). The affinity estimated for the F1760K mutant gave a K_i_ of 2.39 ± 0.28 µM, which turned out to be significantly different compared to that of wildtype channels (*p* < 0.001). From another mutation at this site, it is reported that the affinity decreased 8 to 24-fold for drugs which preferentially interact with the inactivated state (phenytoin, lidocaine) whereas the affinity for the open channel blocker flecainide was weakened only 2–3-fold [36]. Thus, the about 5-fold reduced affinity for aripiprazole at the F1760K mutant ranges at the lower end of this scale. Nevertheless, there is substantial interaction with this site as block development for the wildtype channel required two time constants, while for that of the mutant receptor one time constant was sufficient. Altogether, it remains open which role the local anesthetic binding site plays for the interaction of aripiprazole with the hNa_v_1.5 channel.

## 3. Discussion

Aripiprazole is an atypical antipsychotic drug, which may be used in the treatment of schizophrenia or severe manic episodes in bipolar disorder [4]. Dopaminergic and serotonergic receptors were initially regarded as the primary targets [37]. Later on, it has become evident that aripiprazole interacts to a minor extent with many other metabotropic receptors [13]. Here, we report that aripiprazole is also a potent blocker of VGSCs of the heart muscle type (hNa_v_1.5). The interaction happens in a state-dependent manner, being strongest with the inactivated and open state, with affinities in the sub- to low micromolar concentration range. Aripiprazole reveals no sign of use-dependency and only interacts weakly with the well-known binding site for local anesthetics.

So far, with the exception of one report about potassium channels, no electrophysiological investigations about a possible impact of aripiprazole on voltage-gated ion channels have been conducted [24]. This contrasts with many other antipsychotic agents, which attract attention to these kinds of receptors/channels by entailing cardiotoxic side effects [38,39,40]. For a long time, an interaction of aripiprazole with relevant ion channels of the heart has been disregarded as it has been rated safest among atypical antipsychotic agents with respect to a possible QT prolongation [41]. A similar outcome has been reported from another epidemiological study in which aripiprazole has been classified as a low-risk antipsychotic, at least in healthy patients. The same study even further suggests that aripiprazole might possess only a low affinity for fast VGSCs [20]. This strikingly contrasts to earlier observations in which aripiprazole was investigated for possible neuroprotective properties (see next chapter and [42]). Meanwhile, evidence is provided for the potential of cardiac rhythm disturbances during aripiprazole therapy and in case of overdose [21,29]. Thus, the base for a profound investigation for a possible interaction of aripiprazole with relevant ion channels of the heart muscle was provided. We committed once more on VGSCs, as we recently identified atomoxetine, another antipsychotic drug, as a potent blocker of this channel type [32]. Our data clearly indicate that the affinity of aripiprazole toward the hNa_v_1.5 channel is strong enough to effectively interact with this channel at concentrations occurring in plasma of treated patients [43]. Hence, the question arises which parameters of a drug-channel interaction are relevant to generate adverse side effects?

Another general matter of discussion is the calculation of kinetic parameters. In order to estimate affinity constants from drug-induced changes in kinetic parameters, mathematical procedures have been developed as outlined by Equations (8)–(10). However, this routine has so far only been applied when the time course of block development has been described with a single exponential function. Nevertheless, our data clearly indicate that two time constants are required for a proper description of the drug-induced time courses, at least when higher concentrations of aripiprazole are applied. Therefore, we had the choice either to restrict the evaluation to a mere qualitative description of this behavior or to provide a first, approximate quantitative estimation under the assumption that the data of the two terms might be handled separately as conducted in the present paper. Future studies should develop and evaluate a new model, which will describe the data in scenarios like the one here more precisely by considering the observed changes as a whole.

### 3.1. Side Effects—Pharmacological Safety

So far, several criteria are discussed possibly contributing to a favorable side effect profile of a drug when interacting with a VGSC. First, the interaction should happen in a state-dependent manner with a strong discrimination between the resting and inactivated or open state [44]. This clearly applies to aripiprazole. There is only a low affinity for the resting state, whereas the affinity for the inactivated state is in the submicromolar range, whereby a discrimination factor of about 100 results. Thus, aripiprazole fulfills one favorable criterion concerning the “safety margin” of a VGSC blocker [44]. For a critical comment on the term “resting state”, see our previous manuscript [45]. Overall, our data confirm an otherwise expected outcome from its chemical properties. In this way it is stated that drugs with a high logP value (octanol:water distribution coefficient) and a low pK_a_ value such as riluzole or ritanserin reveal a highly state-dependent behavior [46].

Next, kinetic parameters, especially dissociation/recovery times, seem to be of importance with fast time constants being beneficial. In the pharmacology of the heart, antiarrhythmic drugs of class I (sodium channel blocker) are subdivided according to their dissociation kinetics [47]. Drugs of the subclass Ic are characterized by a rather slow dissociation time (>10 s). A well-known representative thereof is flecainide, revealing a restricted range of applications [48]. Similarly, two local anesthetics (bupivacaine, lidocaine) were graded as either cardiotoxic or cardioprotective due to their slow and fast recovery time constants, respectively. In that report, cardiotoxicity of bupivacaine was ascribed to the slow recovery leading to arrhythmia due to an accumulation of channel block upon repeated activations [49]. Even though a slow recovery of VGSCs from a drug interaction is appraised as unfavorable, inversely a fast recovery is not sufficient to be rated as favorable. In case of antiemetics, both, the fast and slowly recovering metoclopramide and domperidone reveal cardiotoxic effects. Here, VGSCs seem to be of minor importance as cardiotoxicity was explained by the interaction with other ion channels such as hERG [50]. Thus, even though cardiotoxicity can often be linked to an interaction with VGSCs, other targets have also to be considered. Altogether, hNa_v_1.5 channels recover fast from the treatment with aripiprazole whereby no adverse side effects might be expected from this interaction. Whether the newly described side effects upon the application of aripiprazole are due to its interaction with VGSCs or via other targets remains unanswered.

Neuropsychiatric drugs that inhibit VGSCs do this generally in a state-dependent manner though to a different degree. This contrasts to the occurrence and the extent of a use-dependent behavior, which is more variable [27]. Aripiprazole belongs to the rare group that does not operate in a use-dependent manner. Thus, the question arises, what is the impact of use-dependency on cardiac safety? At first glance, an existing use-dependency seems to be favorable as high-frequency events resulting from excessive excitation would be affected to a higher degree than regular activity [51]. However, such a general statement may apply more to neuronal targets. Considering cardiac activity, the blockade of the hNa_v_1.5 channel bears a risk for arrhythmia. The reason for this resides mainly in the propagation velocity of the excitation within the heart. An important aspect here is the rise time of the heart muscle action potential, which on its part correlates with the magnitude of current flow through hNa_v_1.5 channels. Thus, inhibition of this channel can lead to a conduction delay, resulting in a widening of the QRS complex with corresponding consequences [52]. A prolongation of the QRS complex can be observed starting from current reductions by 10% [53]. Such a situation can be fulfilled even at free plasma levels which are 15-fold below the estimated IC_50_ of expressed sodium channels [54]. However, it has also to be considered that use-dependent block can be less in sodium channels of cardiomyocytes compared to heterologously expressed channels [50]. Nevertheless, cardiotoxic adverse effects of flecainide and ranolazine are attributed among other things to their use-dependent inhibition of peak sodium currents [55].

### 3.2. Other Clinical Implications

The inhibition of VGSCs by aripiprazole may also be of importance in other fields, such as neuroprotection or tumor therapy. In this context, aripiprazole is reported to protect cortical neurons from glutamate toxicity [56]. However, the neuroprotective effects of aripiprazole could not be ascribed to its interaction with dopamine D_2_-receptors. A similar outcome was reported from another study [16]. The mode of interaction remained speculative in both cases. In an earlier report, aripiprazole-mediated neuroprotection was indirectly linked to an interaction with VGSCs [42]. Accordingly, aripiprazole suppressed 4-aminopyridine-evoked glutamate release from nerve terminals, which was associated with a diminished influx of sodium ions. Glutamate release was affected only if it was based on neuronal activity, whereas basal, unstimulated release was unaffected. The link to the interaction via sodium channels was finally confirmed as the action of veratridine, an opener of VGSCs was also suppressed by aripiprazole [42]. A more recent overview about neuroprotective effects of second-generation antipsychotics is provided elsewhere [18].

Finally, anticancer activity of aripiprazole with a possible involvement of sodium channels will be considered. Epidemiological studies show that the incidence of cancer is lower in patients suffering from schizophrenia than in healthy ones [19]. Based on this observation, a variety of psychiatric drugs including aripiprazole have been identified to exert anticancer activity on brain tumors. Thus far, many different mechanisms have been proposed to be responsible for this property. The list includes among others an impact on cell proliferation, migration, invasion, or differentiation [19]. In another report, the anticancer property of aripiprazole was explained by its ability to resensitize drug-resistant cancer cells [57]. Conspicuously, all these drugs have in common that they operate as blockers for VGSCs as well. Meanwhile, anticancer properties of sodium channel blockers are well established [30,31]. Thus, the ability of aripiprazole to be beneficial in cancer therapy might also be based on its sodium channel blocking activity.

## 4. Methods

### 4.1. Cell Culture

The TsA201 cell line is a derivative of the human embryonic kidney cell line HEK-293 (ATCC#CRL1537) that expresses a T-antigen against the simian virus 40 (SV40). TsA201 cells were cultured at 37 °C in a humidified atmosphere at 95% air and 5% CO_2_ in DMEM (Dulbeco’s Modified Eagle Medium with 4.5 g/L D-glucose and 2 mM L-glutamine) supplemented with 50 U/mL penicillin, 50 μg/mL streptomycin, and 10% fetal calf serum (Gibco, Eggenstein, Germany). Cells were grown on polyornithine-coated culture dishes to 80% confluence and transfected using the jetPEI DNA transfection reagent (Polyplus, Illkirch, France). The construction of the plasmid pTSV40G-hNa_v_1.5 encoding wild-type hNa_v_1.5 was described previously [58]. This plasmid allows for the simultaneous production of EGFP from a separate expression cassette, and thus for the selection of transfected cells. Mutant channels (hNa_v_1.5_CW and hNa_v_1.5_F1760K) were obtained by respectively modified oligonucleotides and overlapping PCR using a thermostable DNA polymerase with proofreading activity (Pfu DNA Polymerase, Promega GmbH, Germany). The PCR fragments were inserted into the pTSV40G-hNa_v_1.5 background using the restriction sites Age/BsaBI (for CW) and BstEII/SpeI (for F1760K), resulting in pTSV40G-hNa_v_1.5_CW and pTSV40G-hNa_v_1.5_F1760K. Cloning was carried out in the *E. coli* SURE strain (Agilent Technologies, Santa Clara, CA, USA). The correctness of all constructs was confirmed by restriction analysis and, in the case of PCR-derived sequences, by DNA sequencing (Seqlab Microsynth, Göttingen, Germany).

### 4.2. Electrophysiology

Electrophysiological experiments were performed as previously described [32,33,59]. Briefly, TsA201 cells were used for experiments 24 h after transfection. In order to obtain single cells, transfected cells were treated for 1–2 min with TrypLE. Membrane currents were recorded in the whole-cell recording mode using an EPC-9 amplifier and Patchmaster software (v2 × 73; HEKA, Lambrecht, Germany; [60]). Before recording, cells were rinsed twice with an extracellular standard solution containing (in mM): 140 NaCl, 5 KCl, 1.5 CaCl_2_, 1 MgCl_2_, 10 glucose and 12 HEPES (4-(2-hydroxyethyl)piperazine-1-ethanesulfonic acid, N-(2-hydroxyethyl)piperazine-N′-(2-ethanesulfonic acid); pH 7.3). Patch pipettes were drawn from borosilicate glass with tip resistances of about 1.5–2 MΩ, resulting in a typical series resistance <5 MOhm when filled with (in mM): 125 CsF, 10 NaCl, 10 EGTA (ethylene glycol-bis(2-aminoethylether)-*N*,*N*,*N*′,*N*′-tetraacetic acid), 10 HEPES; pH 7.2. To improve sealing, tips were briefly dipped into 2% dimethylsilane dissolved in dichloromethane.

After establishing the whole-cell configuration, the cells were kept for 5 min at the holding potential of −140 mV before data acquisition was initiated. All experiments started with the establishment of a fast inactivation curve (inactivation time 500 ms). Cells with a midpoint of the inactivation curve positive to −80 mV were discarded as these cells might behave differently in pharmacological investigations [61]. Data were filtered by two built-in Bessel filters of the recording system (30 kHz, 6-pole; 10 kHz 4-pole) and sampled at 20–50 kHz. All experiments were conducted at room temperature (22–25 °C).

In order to minimize voltage errors, the series resistance was compensated up to 80%, resulting in voltage errors <6 mV. Leakage currents were subtracted by the P/4 method. No correction for liquid junction potentials was performed. Specific protocols are illustrated in figure legends where appropriate.

### 4.3. Drug Application

The medium in the dish (1.5 mL) was continuously exchanged at a rate of 4.5 mL/min. Reagents were applied locally to the cells by the L/M-SPS-8 superfusion system (List, Darmstadt, Germany). Switching between the eight channels of the superfusion system was controlled by magnetic valves. The local inlet (tip of an eight-barreled pipette) was positioned at a distance of 50–100 μm upstream and the local outlet at about 300 μm downstream of the patch pipette. A constant flow rate of control and test solutions (1 mL/min) was achieved by means of a pressure control system (MPCU-3, Lorenz, Göttingen, Germany). The time of solution exchange was estimated from the changes in the liquid junction potential to be about 1 ms. If not otherwise stated, drugs were applied for at least 10 s before starting the experiments.

### 4.4. Chemicals

Dulbecco’s Modified Eagle Medium, penicillin/streptomycin, and TrypLE Express and fetal calf serum were purchased from Gibco BRL, Eggenstein, Germany. Poly-L-ornithine was purchased from Sigma-Aldrich, Schnelldorf, Germany. Aripiprazole was obtained from Research Chemicals (Toronto, ON, Canada). All other chemicals were obtained from Sigma-Aldrich Chemie GmbH, Steinheim, Germany.

### 4.5. Data Analysis and Statistics

(A) Concentration-inhibition curves for the estimation of half-maximal effective concentrations (IC_50_) were fit to the Hill equation:

(1) $$\frac{I_{A}}{I_{C}}=\frac{1}{1+{\left(\frac{\left[A\right]}{{\mathrm{IC}}_{50}}\right)}^{n}}$$

I_A_ and I_C_ are the current amplitudes in the presence and absence of aripiprazole (A) with [A] representing the concentration of aripiprazole. IC_50_ represents the concentration of aripiprazole that causes 50% inhibition and n is the Hill coefficient.

(B) Voltage dependence of activation

Voltage dependence of activation was calculated in two steps: first, changes in driving force owing to the different test potentials were considered by calculating the conductance g according to:

(2) $$g\;=\frac{I}{V-E_{\mathrm{Na}}}$$

Thereafter, normalized data were fitted with a Boltzmann equation of the form:

(3) $$\frac{g}{g_{\max}}=\frac{1}{1+e^{\left(\frac{V_{50}-\;V}{k}\right)}}$$

(C) Voltage dependence of inactivation

Inactivation is classified as fast or slow, depending on the duration the channels spent in the inactivated state. Voltage dependence of fast inactivation was fitted using a Boltzmann equation of the form:

(4) $$\frac{I}{I_{\max}}=\frac{1}{1+{\;e}^{\left(\frac{V\;-{\;V}_{50}}{k}\right)}}$$

In case of slow inactivation, Equation (4) was extended by the additional parameter, (S) which considers the steady-state level of incomplete inactivation.

(5) $$\frac{I}{I_{\max}}=\left(1-S\right)\left(\frac{1}{1+{\;e}^{\left(\frac{V-V_{50}}{k}\right)}}\right)+\;S$$

V and V_50i_ are actual membrane potentials and the potentials at half-maximal conductance or inactivation, respectively. E_Na_ indicates the reversal potential for sodium ions, which was individually determined for each cell. The slope factor is given by k.

(D) Interaction with the inactivated state

As the inactivated state is non-conducting, the affinity toward this state cannot be measured directly. Therefore, a variety of indirect approaches has been developed as a surrogate.

(1) Shift of inactivation curves/midpoints

From data fitting with Equations (4) and (5), so-called inactivation curves result. The most important parameter here is the potential at which half of the channels are in the inactivated state (V_50_), also called inactivation midpoint. If a drug exerts an inhibitory interaction via an interaction with the inactivated state, inactivation midpoints will be shifted in a concentration-dependent manner to more negative potentials. Thus, from the amount of the shift, the affinity to the inactivated state can be calculated via Equation (6).

(6) $${\Delta V}_{50}=\;k*\ln\left(\frac{1+\frac{\left[A\right]}{K_{r}}}{1+\frac{\left[A\right]}{K_{i}}}\right)$$

K_r_ and K_i_ are the affinities for the resting and inactivated state, respectively. Other abbreviations have the same meaning as before.

(2) Estimation of apparent binding constants

The affinity to the inactivated state (K_i_) can also be calculated from the investigation of partially inactivated channels according to [62], as:

(7) $$\frac{1}{K_{\mathrm{app}}}=\frac{h}{K_{r}}+\frac{\left(1-h\right)}{K_{i}}$$

K_app_ is the apparent affinity estimated at a selected membrane potential at which the amount of non-inactivated channels is given by h (estimated from the corresponding inactivation curve). For K_r_, the experimentally estimated value of 54 µM was used.

(3) Time- and concentration-dependent development of block

The time constant (τ) of block development for the different concentrations of aripiprazole was estimated by double exponential fits of the form:

(8) $$\frac{I}{I_{\max}}=\;S+a_{1}{*e}^{\left(\frac{-t}{\tau_{1}}\right)}+a_{2}{*e}^{\left(\frac{-t}{\tau_{2}}\right)}\;$$

The time constants are given by τ_i_ and the time of inactivation by t.

Association and dissociation rates:

From the time constants of block development, association and dissociation rates can be estimated. To this end, the inverse of the time constants is plotted versus the concentration of aripiprazole. Data were fitted with a linear function:

(9) $$\frac{1}{\tau }={\;k}_{\mathrm{off}}+{\;k}_{\mathrm{on}}*\left[A\right]$$

Estimates for slope and y-intercept give association (k_on_ in µM^−1^s^−1^) and dissociation rate constants (k_off_ in s^−1^) according to [63].

From the rate constants for association and dissociation, the affinity to the inactivated state was calculated as follows:

(10) $$K_{i}=\frac{k_{\mathrm{off}}}{k_{\mathrm{on}}}$$

(E) Recovery from inactivation

Recovery time constants were estimated from normalized data with double or triple exponential functions according to:

(11) $$\frac{I}{I_{\max}}=1-{\;a}_{1}{*e}^{\left(\frac{-t}{\tau_{1}}\right)}-{\;a}_{2}{*e}^{\left(\frac{-t}{\tau_{2}}\right)}-{\;a}_{3}{*e}^{\left(\frac{-t}{\tau_{3}}\right)}$$

Variable identifiers have the same meaning as before.

All curve-fitting procedures were performed using SigmaPlot 13.0 (Sysstat, San Jose, CA, USA). If not directly stated by the presence of error bars, graphs show representative data from single cells. Average values from N = 5 cells are given as mean ± SD in the results section and in figure legends:

### 4.6. Statistics

The effect of aripiprazole on the voltage dependence of activation was tested for statistical significance at a drug concentration of 1 µM by extending Equation (3). The voltage of half-maximal activation V_50_ was modeled to depend linearly on sweep number (to control for linear drifts) and drug exposure. Effects were considered statistically significant if the hypothesis of corresponding parameter values being equal to zero had to be rejected at a significance level of 0.05.

Differences in dissociation constants between WT and F1760K mutant channels were tested for statistical significance by applying Welch’s two sample t-test. Results were considered statistically significant for *p*-values below 0.05.

## 5. Conclusions

Aripiprazole is a potent blocker of the VGSC hNa_v_1.5. Due to the profound analysis of the interaction mechanism, no properties were found which can be made responsible for cardiotoxic side effects. Moreover, the sodium channel-blocking property of aripiprazole might contribute to and also partly explain its neuroprotective and anticancer activity profile.

## Data Availability

The raw data supporting the conclusions of this article will be made available by the authors upon reasonable request.
